# Early detection of Parkinson’s disease from multiple signal speech: Based on Mandarin language dataset

**DOI:** 10.3389/fnagi.2022.1036588

**Published:** 2022-11-10

**Authors:** Qiyue Wang, Yan Fu, Baiyu Shao, Le Chang, Kang Ren, Zhonglue Chen, Yun Ling

**Affiliations:** ^1^School of Mechanical Science and Engineering, Huazhong University of Science and Technology, Wuhan, China; ^2^HUST-GYENNO CNS Intelligent Digital Medicine Technology Center, Wuhan, China; ^3^Gyenno Science Co., Ltd., Shenzhen, China

**Keywords:** Parkinson’s disease, early detection, dysarthria, dysphonia features, machine learning, fully automatic detection model

## Abstract

Parkinson’s disease (PD) is a neurodegenerative disorder that negatively affects millions of people. Early detection is of vital importance. As recent researches showed dysarthria level provides good indicators to the computer-assisted diagnosis and remote monitoring of patients at the early stages. It is the goal of this study to develop an automatic detection method based on newest collected Chinese dataset. Unlike English, no agreement was reached on the main features indicating language disorders due to vocal organ dysfunction. Thus, one of our approaches is to classify the speech phonation and articulation with a machine learning-based feature selection model. Based on a relatively big sample, three feature selection algorithms (LASSO, mRMR, Relief-F) were tested to select the vocal features extracted from speech signals collected in a controlled setting, followed by four classifiers (Naïve Bayes, K-Nearest Neighbor, Logistic Regression and Stochastic Gradient Descent) to detect the disorder. The proposed approach shows an accuracy of 75.76%, sensitivity of 82.44%, specificity of 73.15% and precision of 76.57%, indicating the feasibility and promising future for an automatic and unobtrusive detection on Chinese PD. The comparison among the three selection algorithms reveals that LASSO selector has the best performance regardless types of vocal features. The best detection accuracy is obtained by SGD classifier, while the best resulting sensitivity is obtained by LR classifier. More interestingly, articulation features are more representative and indicative than phonation features among all the selection and classifying algorithms. The most prominent articulation features are F1, F2, DDF1, DDF2, BBE and MFCC.

## Introduction

In China, the incidence rate of Parkinson’s disease (PD) is 1.7% in the population over 65 years old. There are nearly 3 million patients with Parkinson’s disease (PWP) in China, accounting for half of the total number of PWP in the world, and about 100,000 new patients are diagnosed every year. Currently there is no medical cure for PD, but if patients receive timely diagnosis and treatment at the early stage of the disease, early intervention can be applied to delay the disease progress and safeguard daily lives (Singh et al., 2007). Clinical studies have shown that PWP often show some characteristic speech disorders in the early stage (Ho et al., 1999). In 1970, Darley et al. (1969) first studied the pronunciation characteristics of PWP, and found that PWP usually have low volume, increased breath sound, single tone hoarseness and other problems, indicating speech is a useful signal for distinguishing PWP from healthy people (Little et al., 2009; Sapir et al., 2010). Speaking is a highly complex movement that requires the coordination of many nerves and muscles. PWPs commonly have motor deficits, mainly involving the oral, pharyngeal and jaw muscles. Laryngeal and vocal cord tremor, asymmetrical vocal cord closure time, jaw joint dyskinesia and respiratory disorders all lead to voice tremor, unclear speech, slowed speech rate and sunken intonation. At present, there are four main groups of language features used to detect PWP: phonatory, articulatory, prosodic and cognitive-linguistic (Moro-Velazquez et al., 2021).

Phonatory features model abnormal patterns in the vocal fold vibration, whose features were extracted mainly from sustained vowels. Phonation in PWP is characterized by bowing and inadequate closure of vocal folds (Hanson et al., 1984). Articulation deficits in PD patients are mainly associated with reduced amplitude and speed of lip, tongue and jaw movements (Ackermann and Ziegler, 1991), as a result of delayed movements of their tuning organs and a stiff and inflexible tongue, whose features were extracted mainly from running speech (Orozco-Arroyave et al., 2016; Kuruvilla-Dugdale et al., 2020). Prosodic features are paralinguistic, such as pitch variation or the representation of emotions among others (Harel et al., 2004). The cognitive-linguistic analysis examines the vocabulary, phrase construction and the existence of word repetitions (Illes et al., 1988). Among the four types of features, phonation and articulation features are better obtainable, with good constancy to Unified Parkinson’s Disease Rating Scale (UPDRS) and thus most applied in speech analysis on PWP (Dromey et al., 1995; Tykalova et al., 2017; Moro-Velazquez et al., 2021).

PD causes abnormal vocal fold vibration, which can be reflected by the presence of noise and other perturbations caused by incomplete closure (Rusz et al., 2011), abnormal phase closure and phase asymmetry or vocal tremor (Perez et al., 1996). Dysfunction measures including noise or frequency and amplitude perturbations are applied to assess the severity of PD in telemonitoring situations (Little et al., 2009; Tsanas et al., 2010, 2014). The problem is that recordings are done in noisy environment and different equipment draw in different noise, thus affecting dysphonia features being extracted. Studies by Novotny (Novotny et al., 2014) indicate that imprecise consonant articulation can indicate PD-related symptoms. However, it used DDK speech only, which restrict the possible articulatory combinations. Other works employ frequency features, namely Mel Frequency Cepstrum Coefficients (MFCC) and Band Bark Energies (BBE) from running speech, and other features obtained after the segmentation of specific regions, providing good results (Orozco-Arroyave et al., 2016). It is evidenced that the speech of PWP has lower values of relative fundamental frequency, which is the ratio between the fundamental frequency in the cycles of a vowel before or after a voiceless consonant and the typical fundamental frequency during the utterance (Little et al., 2009; Sapir et al., 2010). Other studies perform the tracking of vowel formants during articulation, including onset and offset (Skodda et al., 2012; Bang et al., 2013; Whitfield and Goberman, 2014) and found that as formants reflect the position of the tongue, a reduction of the articulation ranges could subsequently limit the frequency ranges of the formants.

A comparison of PD detection techniques is performed using the acoustic materials extracted from sustained vowels and running speech test, proving that two acoustic materials have better detection performance than utilizing sustained vowels only (Rusz et al., 2013); Bocklet et al. (2013) used phonatory, prosodic and articulatory features jointly, yielding results of 80% of accuracy in PD detection. In any case, all study efforts focus on identify the most representative features for PWP detection but have not reached agreement. The main features for speech sample classification vary across languages (Eyigoz et al., 2020). Different feature extraction methods and different datasets can also obstruct the unification of features (Karan et al., 2020; Zhang et al., 2021). It is one of the main goals for related studies to reduce the number of features by choosing the most relevant for PWP detection.

To date, the majority of studies examining the key characteristics of hypokinetic dysarthria and their relationship to speech intelligibility have been conducted with speakers of English. However, extending this research to languages other than English is important for both theoretical and clinical reasons. Because acoustic cues that strongly influence intelligibility in PD may vary cross-linguistically, which is vital in assessment and treatment planning (Hsu et al., 2017). At present, the speech signal diagnosis for PD patients in China is still in its infancy. Hsu et al. (2017) made a comparison between Mandarin speakers and English speakers on key features of hypokinetic dysarthria. In 2011, Zhang et al. (2011) verified the feasibility of PD Chinese speech detection. Based on their study (Zhang, 2017; Haq et al., 2019) concentrated on phonetic measurements like vocal perturbation and nonlinear measurements to classify PWP. However, they only focused on the vowel pronunciation of PD patients. Although other studies (Su and Chuang, 2015; Fang et al., 2020; Li et al., 2020) filled in this gap through collecting speech samples from vowel pronunciation and running speech test jointly, most of them focus only on time-variant features like MFCC, etc. So this study also intends to figure out if other proposed features recognized can support accurate classification in Chinese speech. Thus it is a worthwhile approach to implement the detection by integrating the automatic feature selection method in case that the system can identify the best representative features by comparing the best detection results based on the newly collected dataset.

Meanwhile existing studies have proposed many machine learning methods to automatically detect PWP although most are based on the manually selected features. Hazan et al. (2012) chose three F1-F2-based acoustic metrics, Formant Centralization Ratio (FAR), Vowel Articulation Index (VAI) and F2i/F2u (the second formant of vowel *i* divided by the second formant of vowel *u*), using the Support Vectors Machine (SVM) with a radial basis function (RBF) kernel for the classification. The best accuracy reached 94%. Gullapalli and Mittal (2022) used various classifiers like Logistic Regression, SVM, KNN, CNN, Deep Neural Network, Boosting, Bagging, Random Forest, and illustrate a comparison on their accuracies, based on MFCC, JTFA, MDVP and TQTW as main features. To date, feature selection has been successfully used in medical applications, where it cannot only reduce dimensionality and but also help us understand the causes of a disease better (Remeseiro and Bolon-Canedo, 2019). Some studies also applied machine learning methods to feature selection. Lamba et al. (2022) tested several combinations of three feature selection approaches (mutual information gain, extra tree, and genetic algorithm) and three classification algorithms (Naive Bayes, KNN, and Random Forest). The combination of genetic algorithm and Random Forest classifier has shown the best performance with 95.58% accuracy. Solana-Lavalle et al. (2020) used Wrappers feature subset selection with four classifiers (KNN, Multi-layer perceptron, SVM, and Random Forest), obtaining the highest accuracy of 94.7% with SVM. The fully automatic model mentioned above performs well in corpora such as English and German, but it is still unknown whether this kind of model can be well applied to the detection of Chinese PD.

In this study, data are collected from two types of speech tasks (namely sustain vowel sound and running speech test), and a completely automatic model is proposed. Multiple speech signals are extracted and are fed into the hybrid combinations of three feature selectors and four classifiers, detecting PWP automatically. The final detection results are used to compare the performance of both selection algorithms and classifying algorithms. The manuscript is organized as follows. Section “Materials and methods” elaborates the dataset and discusses the automatic PD detection model, with classifier validation methods and evaluation metrics. In section “Results,” experimental results are given in details. Section “Discussion” makes a discussion about the results. Some concluding remarks are given in the section “Conclusion.”

## Materials and methods

### Automatic detection model

**FIGURE 1:**
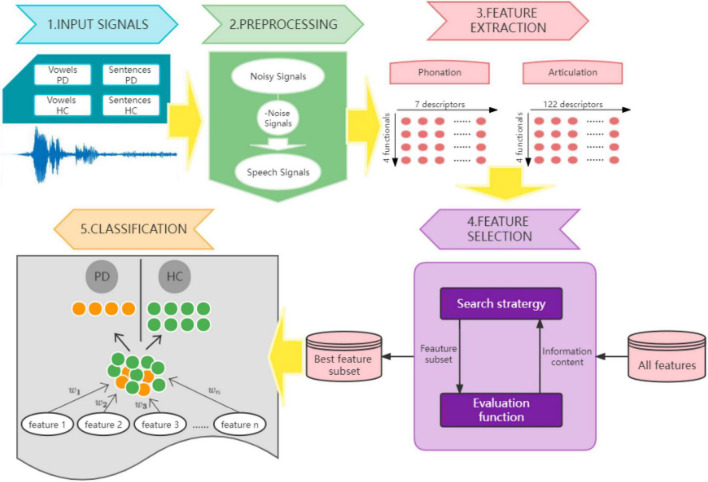
Automatic Parkinson’s disease detection model.

In this study, an automatic detection model is proposed, including feature extraction, feature selection and tester classification, as shown in Figure 1.

When the data are collected, noise reduction is firstly conducted. The noise-reduced speech signal is applied to feature extraction processing, and two types of static vector features are extracted, respectively. The feature extraction is run on the open-source algorithm on GitHub.^1^ For feature selection module, the study chose three algorithms to filter the extracted features to compare the best selection algorithm that can integrated in the proposed model. For feature classifying, four algorithms are tested to determine the best to be embedded in the model.

### Database

Clinical practice has shown that sustained vowels (Tsanas et al., 2014) and running speech (Ackermann and Ziegler, 1991) are good materials for detection. This study uses two corpora for testing analysis: sustained vowels and tongue twisters. Tongue twister is regarded as challenging to pronounce because of meeting problems of using correctly the mouth and tongue. It could be assumed that dysarthria would manifest especially during trying to pronounce togue twister by PD due to the deterioration of articulators (Vilda et al., 2017). Data were collected at National Research Center of Geriatric Diseases, Tongji Hospital under the supervision of SLP professionals. 100 Mandarin-speaking people were recorded. 50 are diagnosed as PD (25 female, 25 male) and 50 healthy people (25 female, 25 male). The 50 PD patients have an average age of 63.57 ± 11.31 years (mean ± SD) and a mean disease duration of 6.08 ± 3.17 years. According to Hoehn and Yahr (HY) staging scale, all patients were in stage 1–3 (1–1.5 as early stage and 2–3 as middle stage). None of them has a history of language or speech disorders. Their mean motor score according to part III of MDS_UPDRS was 36.43 ± 17.39. Each person (50 PWP, 50 HC) was recorded three times for each of four speech samples, and a total of 1,200 speech signals were collected for the dataset. The first two recording tasks are sustained vowels (“aaa…” and “eee…” in Chinese Pinyin), from which the 6-s stable segments are extracted; the other two are short sentences (“si shi si zhi shi shi zi” and “yi zhi da hua wan kou zhe yi zhi da hua ha ma”). The voice recordings have been obtained in a realistic environment using a Rode NT-USB microphone 10 cm away from the mouth. And the sampling rate of data was 96 kHz. Data has been stored in a WAVE (.wav) file format. Sustained vowels were used in the phonatory analysis, while running speech test (sentence 1, sentence 2) was added for articulation analysis. Spectral subtraction (SS) (Boll, 1979) is used to clean up the noisy speech signal.

### Feature selection algorithm

Three algorithms, Least Absolute Shrinkage and Selection Operator (LASSO), minimum-Redundancy-Maximum-Relevance (mRMR), and Relief-F are applied for automatic feature selection. Each of them is applicable to the selection of high-dimensional data sets and has a wide range of applications in many fields. The number of features is set on the random rule. The most representative features can be determined upon comparing the accuracy of the final classification results.

#### Least Absolute Shrinkage and Selection Operator

The LASSO (Fonti and Belitser, 2017), mainly for feature selection of high-dimensional data allows the coefficients of features to be compressed even to zero. Capable of making up for the deficiencies of least squares and stepwise regression for local optimal estimation, it can effectively solve the problem of multicollinearity existing among the features. The LASSO is a particular case of the penalized least squares regression with *L_1_*-penalty function.

The LASSO estimate can be defined by:


(1) $${\hat{\beta }}^{l\,a\,s\,s\,o}=argmin\{\frac{1}{2}\sum_{i=1}^{N}{\left(y_{i}-\beta_{0}-\sum_{j=1}^{p}x_{i\,j}\beta_{j}\right)}^{2}+\lambda \sum_{j=1}^{p}|\beta_{j}|\}$$

LASSO transforms each and every coefficient by a constant component λ, truncating at zero. Hence it is a forward-looking variable selection method for regression. It decreases the residual sum of squares subject to the sum of the absolute value of the coefficients being less than a constant. LASSO improves both prediction accuracy and model interpretability by combining the good qualities of ridge regression and subset selection. If there is high correlation in the group of predictors, LASSO chooses only one among them and shrinks the others to zero. It reduces the variability of the estimates by shrinking the some of the coefficients exactly to zero producing easily interpretable models (Muthukrishnan and Rohini, 2016).

#### Minimum-Redundancy-Maximum-Relevance

Minimum-Redundancy-Maximum-Relevance (mRMR) algorithm (Solana-Lavalle et al., 2020) is a typical feature selection algorithm based on spatial search. It extracts features of maximum relevance to the target variable while ensuring minimum redundancy between each other. In this algorithm, both redundancy and correlation are used as the metric of mutual information. The steps involved are:

1. Calculate the mutual information of each special *x_i_* with category *C*:


(2) $$\begin{array}{c} I\,\left(x_{i};C\right) \end{array}$$

2. The average of the mutual information between all features and the category *C* is calculated to obtain an approximation of *D*. A subset *S*of features containing *m* features is drawn so that the value of *D* calculated using the features within *S* is maximized:


(3) $$m\,a\,x\,D\,\left(S;C\right)\,D=\frac{1}{\left|S\right|}\,\sum_{x_{i}\in S}I\,\left(x_{i};C\right)$$

3. Eliminate the redundancy between the selected *m* features:


(4) $$m\,i\,n\,R\,\left(S\right)\,R=\frac{1}{{\left|S\right|}^{2}}\,\sum_{x_{i},x_{j}\in S}I\,\left(x_{i};x_{j}\right)$$

4. Calculate set *S* of features with maximum-relevance-minimum-redundancy:


(5) $$\text{mRMR}=\text{max}\,\left[\frac{1}{\left|\text{S}\right|}\,\sum_{x_{i}}I\,\left(x_{i};C\right)-\frac{1}{{\left|S\right|}^{2}}\,\sum_{x_{i};x_{j}\in S}I\,\left(x_{i};x_{j}\right)\right]$$

#### Relief-F

Relief-F (Park and Kwon, 2007), as the more effective filter-style feature evaluation algorithm is proposed for regression problems where the target attributes are continuous values. Relief algorithm assigns each feature weights, subsequently updated. Features with higher correlation with labels are given higher weights, and vice versa. The steps involved are:

1. Let the training data set be D, the number of samples sampled be m, the feature weight threshold be δ, and the number of nearest samples be k, the feature weights of each characteristic of the output be T.

2. Set all feature weights to zero and make T the empty set.

3. For *i* = 1,2,⋯m: (a) Select a random sample *R*from *D*; (b) finds *k* nearest-neighbor samples H_j_(j=1,2,⋯k) of R from the sample set of the same category, and *k* nearest-neighbor samples*M*_*j*_(*C*) of *R*from the sample set of different categories.

4. For *A* = 1*to*N. All features do:


(6) $$W\,\left(A\right)=W\,\left(A\right)-\sum_{j=1}^{k}\frac{d\,i\,f\,f\,\left(A,R,H_{j}\right)}{m\,k}+$$

$$\sum_{C\,e\,c\,i\,a\,s\,s\,\left(R\right)}\frac{\frac{p\,\left(C\right)}{1-p\,\left(C\,l\,a\,s\,s\,\left(R\right)\right)}\,\sum_{j=1}^{k}d\,i\,f\,f\,\left(A,R,M_{j}\,\left(C\right)\right)}{m\,k}$$

### Classifier

Four classifying algorithms, Naive Bayes, K-Nearest Neighbor (KNN), Logistic Regression and Stochastic Gradient Descent are trained by the dataset to explore the best for the whole model.

#### Naïve Bayes

The input space vector *𝒳* ⊆ ^*Rn*^ is the set of n-dimensional vectors and the output space vector is the set of class labels *𝒴* = {*c*_1_,*c*_2_,⋯,*c*_*k*_}. The input is the feature vector *x* ∈ *𝒳* and the output is the class label y ∈ *𝒴*. *X* is a random vector defined on the input space *𝒳*, and *Y* is a random variable defined on the output space *𝒴*. *P*(*X*,*Y*) is the joint probability distribution of *X* and *Y*.

In the Naïve Bayes, for a given input *x*, the posterior probability distribution *P*(*Y* = *c*_*k*_|*X* = *x*) is calculated by the learned model, and the class with the highest posterior probability is used as the class output of *x*. The posterior probability calculation is performed according to Bayes’ theorem. Finally, by the substitution calculation of the formulars, the Naive Bayesian classifier can be expressed as:


(7) $$y=\text{arg}\text{maxP}\left(\text{Y}={\text{c}}_{\text{k}}\right)\prod_{\text{j}}\text{P}\left({\text{X}}^{\left(\text{j}\right)}={\text{x}}^{\left(\text{j}\right)}|\text{Y}={\text{c}}_{\text{k}}\right)$$

#### K-Nearest Neighbor

K-Nearest Neighbor (KNN) assumes a given training dataset in which the strength classes have been determined. New instances are predicted based on the categories of their k-nearest neighboring training instances, e.g., by majority voting. KNN does not have an explicit learning process, but uses the training dataset to partition the feature vector space and serve as a “model” for its classification. The core idea of the algorithm is that a sample belongs to a class if most of its k-nearest samples belong to that class. And the measurement of distance generally adopts the Euclidean distance:


(8) $$\text{d}\,\left(\text{x},\text{y}\right)=\sqrt{\sum_{\text{k}=1}^{\text{n}}{\left({\text{x}}_{\text{k}}-{\text{y}}_{\text{k}}\right)}^{2}}$$

#### Logistic Regression

The LR algorithm is a typical and mature classification algorithm, which performs well especially in binary classification problems. Since speech data have many features, each of which has certain level of influence on the final classification result and needs to be linearly weighted. The output of LR is not the exact category, but a probability, and if the result is closer to 0 or 1, the higher the confidence of the classification result is higher. Weighting of each feature can be adjusted by the classification result during the training process, making the classification result more accurate.

Regression routine steps are as follows:

(1) Find the prediction function.


(9) $$h_{\theta }\,\left(x\right)=g\,\left(\theta^{T}\,x\right)=\frac{1}{1+e^{-\theta^{T}\,x}}$$

The value of *h*_θ_(*x*) indicates the probability that the result will take 1. For input *x*, the probability that the classification results in category 1 and category 0, respectively, are:


(10) $$P\left(y=1|x;\theta \right)=h_{\theta }\left(x\right)$$

(11) $$P\left(y=0|x;\theta \right)=1-h_{\theta }\left(x\right)$$

(2) Find the loss function.

The Cost-function and J-function are as follows, and they are derived based on the maximum likelihood estimation.


(12) $$C\,o\,s\,t\,\left(h_{\theta }\,\left(x\right),y\right)=\left\{ \begin{array}{cc} -l\,o\,g\,\left(h_{\theta }\,\left(x\right)\right)\; & y=1\\ -l\,o\,g\,\left(1-h_{\theta }\,\left(x\right)\right)\; & y=0 \end{array}\right.$$

(13) $$J\,\left(\theta \right)=\frac{1}{m}\,\sum_{i=1}^{m}C\,o\,s\,t\,\left(h_{\theta }\,\left(x_{i}\right),y_{i}\right)=-\frac{1}{m}$$

$$\left[\sum_{i=1}^{m}\left(y_{i}\,l\,o\,g\,h_{\theta }\,\left(x_{i}\right)+\left(1-y_{i}\right)\,l\,o\,g\,\left(1-h_{\theta }\,\left(x_{i}\right)\right)\right)\right]$$

(3) Minimize the loss function and find the regression parameter θ.


(14) $$\theta_{j}:=\theta_{j}-\alpha \frac{1}{m}\sum_{i=1}^{m}\left(h_{\theta }\left(x_{i}\right)-y_{i}\right)x_{i}{}^{j}$$

#### Stochastic Gradient Decent

An arbitrary hyperplane *w_0_*, *b_0_* chosen and then the objective function is continuously minimized using Stochastic Gradient Descent. Assuming that the set of misclassified points *M* is fixed, the gradient of the loss function *L*(*w*,*b*) is as follows:


(15) $$\nabla_{w}L\,\left(w,b\right)=-\sum_{x_{i}\in M}y_{i}\,x_{i}$$

(16) $$\nabla_{b}L\,\left(w,b\right)=-\sum_{x_{i}\in M}y_{i}$$

Select a random misclassification point (*x*_*i*_,*y*_*i*_) and update *w*, *b*:


(17) $$w\leftarrow w+\eta \,x_{i}\,y_{i}$$

(18) $$b\leftarrow b+\eta \,y_{i}$$

where η(0 < η≤1) denotes the step size, also known as the learning rate in statistics. The loss function *L*(*w*,*b*) can be reduced by iterations until it is 0, which means that the point is correctly classified.

### Performance metrics

There are four results for the detection: TRUE POSITIVE (TP) if a PD patient is correctly identified and otherwise FALSE NEGATIVE (FN), TRUE NEGATIVES (TN) if healthy subjects correctly diagnosed and otherwise FALSE POSITIVES (FP).

Accuracy, sensitivity, specificity, precision, false alarm rate, Matthew correlation coefficient, F1 score and the receiver operating curve (ROC) are used to make statistical analysis on the results.

Accuracy represents the percentage that the classification is correct.


(19) $$\text{Accuracy}=\frac{\text{TP}+\text{TN}}{\text{TP}+\text{TN}+\text{FP}+\text{FN}}$$

Sensitivity or recall is the probability that the outcome of diagnosing PD is positive given that the subjects have PD.


(20) $$\text{Sensitivity}=\frac{\text{TP}}{\text{TP}+\text{FN}}$$

Specificity represents the proportion that the outcome of PD is negative given that the subject is healthy.


(21) $$\text{Specificity}=\frac{T\,N}{T\,N+F\,P}$$

Precision is the probability that the outcome of diagnosing PD is true.


(22) $$\text{Precision}=\frac{T\,P}{T\,P+F\,P}$$

The false alarm rate (FAR) is the probability that the outcome of diagnosing PD is false.


(23) $$F\,A\,R=\frac{F\,P}{T\,P+F\,P}$$

The Matthews correlation coefficient (MCC) is a correlation coefficient between the observed and predicted binary classifications. It returns a value between –1 and +1. When MCC = 1, it means that machine learning system perfectly predict the category of the object; When the value is 0, it indicates that the predicted result is worse than the random prediction result; When MCC = –1, it illustrates that the predicted classification is completely inconsistent with the actual classification.


(24) $$\text{MCC}=\frac{\text{TP}\times \text{TN}-\text{FP}\times \text{FN}}{\sqrt{\left(\text{TP}+\text{FP}\right)\,\left(\text{TP}+\text{FN}\right)\,\left(\text{TN}+\text{FP}\right)\,\left(\text{TN}+\text{FN}\right)}}$$

The *F_1_* score is the harmonic mean of precision and recall.


(25) $${\text{F}}_{1}=2\,\frac{\text{precision}\times \text{sensitivity}}{\text{precision}+\text{sensitivity}}$$

The receiver operating characteristic (ROC) curve is the plot of the Sensitivity against the false positive rate (*FPR* = 1−*Specificity*) in a binary classifier when its threshold is varied.

### Training and test set

**FIGURE 2:**
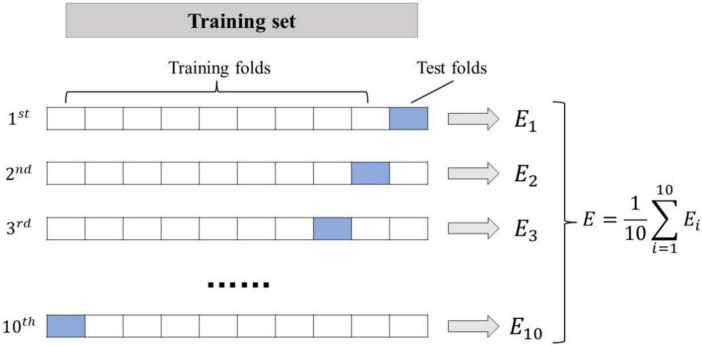
Tenfold cross-validation schematic.

Feature vectors, from PD or HC, are stored into two sets: *C_1_* for PD patients, *C_2_* for HC. Each set is separated into ten fragments, *C*_1_ = {*C*_1,1_,*C*_1,2_,⋯,*C*_1,10_} and *C*_2_ = {*C*_2,1_,*C*_2,2_,⋯,*C*_2,10_}. A fragment *C*_*1,i*_ (from *C_1_*) and a corresponding fragment *C*_*2,i*_ (from *C_2_*) are randomly combined into *C_i_*. The result of these random mixings is tenfold {*C*_1_,*C*_2_,⋯*C*_10_}, where each fold contains instances from PD and HC. Among the ten folds, one is picked for testing of a classifier and the other nine are left for training. (Solana-Lavalle et al., 2020). To be specific, in this study, the speech data with 5 PD and 5 HC are used as the test set, and the speech data with 45 PD and 45 HC are used as the training set. The tenfold cross-validation schematic is shown in Figure 2.

## Results

Table 1 presents the description of the two main types of features extracted from the speech signal and the corresponding number of features within each group.

**TABLE 1:** Features extracted from speech signals.

Feature set	Description	Total features
Phonation	Jitter, Shimmer, APQ, PPQ, DF0, DDF0, LogE.	28
Articulation	BBE_on (22 levels), BBE_off (22 levels); MFCC_on (12 levels), MFCC_off (12 levels); DMFCC_on (12 levels), DMFCC_off (12 levels); DDMFCC_on (12 levels), DDMFCC_off (12 levels); F1 (1 level), DF1 (1 level), DDF1 (1 level); F2 (1 level), DF2 (1 level), DDF2 (1 level).	488

Twenty-eight phonation features were extracted. There are seven descriptors, each of which has four values: mean, standard deviation, skewness, and kurtosis. The seven descriptors are Jitter, Shimmer, Pitch Perturbation Quotient (PPQ), Amplitude Perturbation Quotient (APQ), First Derivative of the Fundamental Frequency (DF0), Second Derivative of the Fundamental Frequency (DDF0), and Logaritmic Energy (LogE).

Four hundred and eighty-eight articulation features were extracted. There are 122 descriptors, each of which has 4 values: mean, standard deviation, skewness and kurtosis. The 122 descriptors are be segmented into 14 categories: Bark Band Energies in onset transitions (BBE_on), Bark Band Energies in offset transitions (BBE_off), Mel Frequency Cepstral Coefficients in onset transitions (MFCC_on), Mel Frequency Cepstral Coefficients in offset transitions (MFCC_off), First derivative of the MFCCs in onset transitions (DMFCC_on), First derivative of the MFCCs in offset transitions (DMFCC_off), Second derivative of the MFCCs in onset transitions (DDMFCC_on), Second derivative of the MFCCs in offset transitions (DDMFCC_off), First Formant Frequency (F1), First Derivative of F1 (DF1), Second Derivative of F1 (DDF1), Second Formant Frequency (F2), First Derivative of F2 (DF2) and Second Derivative of F2 (DDF2). Each category has a different number of levels, as shown in Table 1.

Table 2 presents a description of the selected features. For phonation, each algorithm screens 5, 7, and 12 features for classification each time; for articulation, each algorithm screens 10, 20, 30, and 40 features for classification each time. The best performing feature set and its size corresponding to each algorithm are listed in Table 2.

**TABLE 2:** Features obtained by using three features selection algorithms (std stands for standard deviation).

Feature subset	Features		
	
	LASSO	mRMR	Relief-F
Phonation features	Kurtosis_Jitter, kurtosis_Shimmer, kurtosis_DF0, kurtosis_PPQ, kurtosis_APQ. (5)	Kurtosis_Jitter, mean_Jitter, mean_Shimmer, std_PPQ, skewness_Jitter, std_APQ, skewness_Shimmer. (7)	Std_LogE, skewness_LogE, mean_DF0, kurtosis_Shimmer, mean_LogE, mean_DDF0, skewness_PPQ, skewness_DDF0, kurtosis_APQ, skewness_APQ, skewness_DF0, kurtosis_PPQ. (12)
Articulation features	Mean_MFCCon_3, mean_MFCCon_4, mean_MFCCoff_3, mean_F1, mean_F2, std_F1, std_DDF1, std_F2, std_DDF2, mean_MFCCon_6. (10)	Mean_F2, mean_BBEon_2, mean_BBEoff_1, mean_DDMFCCoff_1, std_F1, std_DDF1, std_DDF2, mean_F1, mean_BBEon_3, std_F2. (10)	Mean_MFCCon_3, mean_MFCCoff_3, mean_BBEoff_6, mean_MFCCon_4, mean_MFCCoff_4, mean_BBEoff_7, mean_F1, mean_MFCCoff_7, mean_F2, mean_MFCCon_7. (10)

Tables 3–5 show the PD detection performance of four different classifiers (Naïve Bayes, KNN, Logistic Regression, Stochastic Gradient Descent) when they are tested with a phonation feature set selected by three selection algorithms (LASSO, mRMR, Relief-F). The best results are highlighted in boldface. The best performance metric values for phonation-based PD detection are Accuracy = 0.4941, Sensitivity = 0.7058, Specificity = 0.8070, precision = 0.5100, FAR = 0.4900, MCC = 0.0413, *F_1_*score = 0.5449. Most these best results appear when the LASSO algorithm is used to select the features. The best-performing classifier is different across different features.

**TABLE 3:** PD-detection performance metrics for four different classifiers by using phonation features selected by Least Absolute Shrinkage and Selection Operator selection algorithm.

	Accuracy	Sensitivity	Specificity	Precision	FAR	MCC	F_1_score
KNN	0.4898	**0.7058**	**0.7058**	**0.4344**	**0.5656**	**0.0413**	**0.5378**
LR	0.3726	0.5167	0.3777	0.3680	0.6320	–0.0837	0.4298
SGD	0.3391	0.4900	0.3400	0.3382	0.6618	–0.1810	0.4002
NB	**0.4941**	0.1483	0.8070	0.1531	0.8469	–0.0330	0.1507

The best results are highlighted in bold.

**TABLE 4:** Parkinson’s disease-detection performance metrics for four different classifiers by using phonation features selected by minimum-Redundancy-Maximum-Relevance selection algorithm.

	Accuracy	Sensitivity	Specificity	Precision	FAR	MCC	F_1_score
KNN	**0.4475**	**0.6501**	**0.6501**	**0.3832**	**0.6168**	**–0.0133**	**0.4822**
LR	0.3184	0.3989	0.3400	0.2906	0.7094	–0.2865	0.3362
SGD	0.3558	0.6544	0.1547	0.1350	0.8650	–0.2110	0.2238
NB	0.3141	0.3628	0.4000	0.2281	0.7719	–0.2290	0.2801

The best results are highlighted in bold.

**TABLE 5:** Parkinson’s disease-detection performance metrics for four different classifiers by using phonation features selected by Relief-F selection algorithm.

	Accuracy	Sensitivity	Specificity	Precision	FAR	MCC	F_1_score
KNN	0.4354	**0.6497**	**0.6497**	0.3697	0.6303	–0.0486	0.4712
LR	0.4351	0.5711	0.4201	**0.4471**	**0.5529**	**–0.0108**	**0.5015**
SGD	0.4143	0.4067	0.5374	0.3105	0.6895	–0.0337	0.3522
NB	**0.4399**	0.4128	0.5222	0.3593	0.6407	–0.0708	0.3842

The best results are highlighted in bold.

Tables 6–8 show the PD detection performance of the four classifiers, when they are tested with an articulation feature set automatically selected. The best results are also highlighted in boldface. The best performance metric values for articulation-based PD detection are Accuracy = 0.7576, Sensitivity = 0.8244, Specificity = 0.7315, precision = 0.7657, FAR = 0.2343, MCC = 0.5100, *F_1_*score = 0.7901. All these best results also appear when the LASSO algorithm is used to select the features.

**TABLE 6:** PD-detection performance metrics for four different classifiers by using articulation features selected by Least Absolute Shrinkage and Selection Operator selection algorithm.

	Accuracy	Sensitivity	Specificity	Precision	FAR	MCC	F_1_score
KNN	0.5017	0.5851	0.5851	0.4556	0.5444	0.1110	0.5123
LR	0.7453	**0.8244**	0.6885	0.7586	0.2414	**0.5100**	**0.7901**
SGD	**0.7576**	0.7717	**0.7315**	**0.7657**	**0.2343**	0.5041	0.7687
NB	0.3981	0.5267	0.4080	0.3896	0.6140	–0.0418	0.4479

The best results are highlighted in bold.

**TABLE 7:** PD-detection performance metrics for four different classifiers by using articulation features selected by minimum-Redundancy-Maximum-Relevance selection algorithm.

	Accuracy	Sensitivity	Specificity	Precision	FAR	MCC	F_1_score
KNN	0.4605	**0.6648**	**0.6648**	0.3987	0.6013	–0.0395	0.4985
LR	0.5063	0.6156	0.4584	0.5416	0.4584	0.0627	**0.5763**
SGD	**0.5734**	0.5706	0.5988	**0.5557**	**0.4443**	**0.1688**	0.5630
NB	0.3266	0.4644	0.3333	0.3192	0.6808	–0.2448	0.3783

The best results are highlighted in bold.

**TABLE 8:** PD-detection performance metrics for four different classifiers by using articulation features selected by Relief-F selection algorithm.

	Accuracy	Sensitivity	Specificity	Precision	FAR	MCC	F_1_score
KNN	0.5482	0.6383	0.6383	0.5076	0.4924	0.1295	0.5655
LR	**0.6994**	**0.7839**	**0.6450**	**0.7160**	**0.2840**	**0.4277**	**0.7484**
SGD	0.6743	0.7217	0.6326	0.6919	0.3081	0.3590	0.7065
NB	0.5534	0.5361	0.6276	0.5020	0.4980	0.1457	0.5185

The best results are highlighted in bold.

## Discussion

For all three selection algorithms, the most prominent features are F1, F2, DDF, BBE and MFCC, which are all from articulation features. F1, F2, DDF1 and DDF2 can represent resonances in the vocal tract (Pah et al., 2022) and the capability of the speaker to hold the tongue in a certain position (Ladefoged and Harshman, 1979). BBE and MFCC are common dynamic signals. It was found that oral rotation can be represented by the dynamic characteristics of speech signals (like BBE and MFCC). Although the oral rotation rate of PWP did not decrease significantly, there was a balance among speed, intensity and accuracy. Besides, MFCCs were also computed as a smooth representation of the voice spectrum that considers the human auditory perception. The features mentioned above may mainly reflect the pitch, speed and intelligibility of the tester’s speech (Moro-Velazquez et al., 2021), echoing UPDRS, which can reflect the five levels of speech status from 0 to 4 in the scale (Zhang et al., 2017).

It is quite noticeable that articulation-type features are generally more representative than phonation analysis in this study. The reason may be that, the signals employed in phonatory approaches (sustained vowels) are much simpler than those used for articulatory analyses (running speech), including less variability and a smaller amount of kinetic information. Moreover, running speech contains vowels and sonorant segments and therefore methodologies using connected speech can indirectly characterize certain phonatory aspects (Moro-Velazquez et al., 2021). In addition, articulation relates to more voice organs than phonation features (Hanson et al., 1984; Ackermann and Ziegler, 1991). Phonation features like Jitter and Shimmer are used as significant influential factors in classifiers, with good performance of the results (Orozco-Arroyave et al., 2014; Mekyska et al., 2015; Moro-Velazquez et al., 2021). But in the present study, all phonation feature subsets provide relatively low classification accuracy. Further studies based on more Chinese dataset are expected to explore the reasons.

**FIGURE 3:**
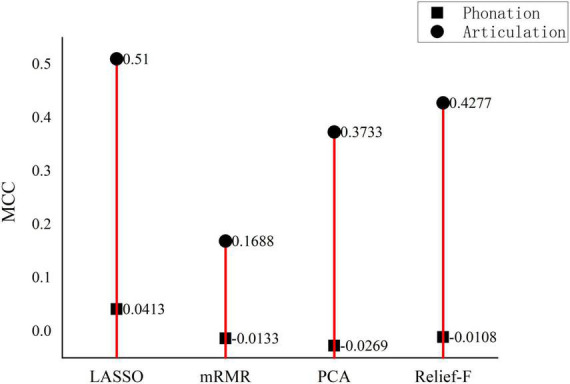
Comparison of Matthews correlation coefficient values from different models.

**FIGURE 4:**
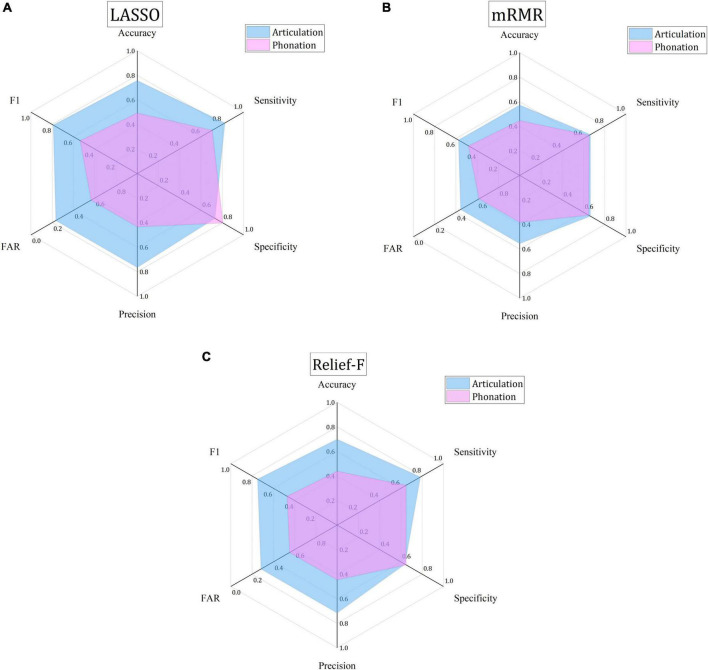
Comparison of the best classification performances based on three FS algorithms and two types of features. **(A)** The best classification performance based on LASSO algorithm. **(B)** The best classification performance based on mRMR algorithm. **(C)** The best classification performance based on Relief-F algorithm.

As Figure 3 shows, the MCC values for the detection with the phonation features are almost all negative small numbers while the MCC for the articulation features are mostly positive big numbers. Therefore, it is recommended that articulation features can the primary detection feature set applied. Further studies can be made to identify the most representative articulation features when more Chinese language materials are used to test the proposed detection model. Six positive performance values are further compared as shown in Figure 4. The articulation (indicated by blue) and phonation (indicated by pink) are put together to show that the articulation feature is better than the phonation feature, indicating articulation can better reflect the phonetic features of Chinese PWP. This finding is quite consistent with the conclusion reached by Vásquez-Correa et al. (2018). It can be inferred that the proposed model can automatically generate good indicators for the following automatic speech character classification, replacing the manual feature selection.

Meanwhile, it was found that the MCC values of the final detection results were not satisfactory enough. There are several possible reasons:Firstly, the speech data used in this study were from patients at HY1-3 stages, with relatively low degree of dysarthria, and pronunciation defects were not quite obvious. Secondly, the classification features are automatically selected by the model, and the number of input features is large, which may result in overfitting and cause some errors. Thirdly, different from English language, each hieroglyph in Chinese has an individual meaning and corresponds to one syllable, which means each syllable conveys an idea, and the combination of syllables will be different according to different contextual meanings. Chinese speakers normally require more time to think before speaking, causing some pauses, not due to PD (Pavlovskaya and Hao, 2020). Chinese speakers breathe less regularly than English speakers when speaking, which in turn may lead to a misjudgment that Chinese test subjects have unstable vocalizations.

Among the three feature selection algorithms, LASSO performs the best. The main reason may lie in that the parameter estimation of LASSO algorithm with good continuity is suitable for the selection model of high-dimensional data, which is the main characters of the collected signal. Among the four classification algorithms, Logistic Regression performs the best. To observe the final performance results, LASSO & LR and LASSO & SGD are the best combinations of feature selection and classifier with the accuracies of 0.7453 and 0.7576, respectively. Interestingly, for English corpus, the commonly used Wrappers feature subset selection and the classifying techniques like KNN, SVM, MLP and Random Forest do not perform well for Chinese corpus in this study. More speech materials should be collected to train the detection model, and comparing the results with the performance of those algorithms in case.

Overall, the results prove the feasibility of applying a fully automatic model to Chinese PD detection is feasible, even though the results are not satisfactory when compared with the detection model based on the English corpus (Solana-Lavalle et al., 2020). But the combined performance of LASSO with LR and SGD are both above 0.7, quite convincing to motivate further development on the proposed detection including automatic feature selection and classification when there are no universally accepted representative features for PWP early detection.

## Conclusion

The novel contribution of this study is establishing an automatic model with machine learning methods based on Mandarin language dataset, dealing the whole process of PD detection based on speech signals from extraction, selection to classifying automatically. It is possible that the gap-filling in setting up representative feature reservoirs for Chinese language can be accelerated through automatic feature selection model.

The current study only proved its feasibility and future work should be focused on developing robust and accurate methods for the automatic and unobtrusive detection for Chinese PWP, and a dedicated algorithm for feature extraction specific to Chinese speech features.

This study also gives good hints for feature selection and classifier strategy. The most representative feature set of PWP is articulation, from which ten features automatically selected is enough for the following classifier. To improve the accuracy of detection, bigger dataset should be collected to test whether the articulation features are the best representative for Chinese-speaking PD. LASSO performs the best feature selection and LR performs the best classification, while two combinations of LASSO & LR and LASSO & SGD all performs well. So, in this study, the best model proposed is to filter 10 articulation features with LASSO algorithm and use them in SGD or LR classifier. Further studies can be made to explore the rules of selecting among LASSO & LR or LASSO & SGD.

## Data availability statement

The raw data supporting the conclusions of this article will be made available by the authors, without undue reservation.

## Ethics statement

Data collection and sharing for this project was approved by the Medical Ethics Committee of Tongji Hospital (A053/IEC/2021). Prior to data acquisition, all patients involved gave written informed consent to the study procedures, and to pseudonymized storage of voice recordings and further speech analyses.

## Author contributions

QW: methodology, software, formal analysis, investigation, writing – original draft and review and editing, and visualization. YF: conceptualization, validation, formal analysis, writing – original draft, supervision, and project administration. BS: data curation, resources, and investigation. LC: software, formal analysis, and writing – review and editing. KR: study design and writing – review the draft. ZC: study design and data collection. YL: study design. All authors contributed to the article and approved the submitted version.

## Footnotes

1
https://github.com/jcvasquezc/DisVoice
